# Explainable lung cancer classification with ensemble transfer learning of VGG16, Resnet50 and InceptionV3 using grad-cam

**DOI:** 10.1186/s12880-024-01345-x

**Published:** 2024-07-19

**Authors:** Yogesh Kumaran S, J. Jospin Jeya, Mahesh T R, Surbhi Bhatia Khan, Saeed Alzahrani, Mohammed Alojail

**Affiliations:** 1 Department of Computer Science & Engineering, Faculty of Engineering and Technology, JAIN (Deemed-to-be University), Bengaluru, 562112 India; 2 Department of Computer Science and Engineering, SRM Institute of Science and Technology, Ramapuram, Chennai India; 3 School of science, engineering and environment, University of Salford, Manchester, UK; 4 Management Information System Department, College of Business Administration, King Saud University, Riyadh, Saudi Arabia

**Keywords:** Deep learning, Medical imaging, Lung cancer detection, VGG16, ResNet50, InceptionV3, SMOTE, Image classification, Feature extraction, Diagnostic accuracy

## Abstract

Medical imaging stands as a critical component in diagnosing various diseases, where traditional methods often rely on manual interpretation and conventional machine learning techniques. These approaches, while effective, come with inherent limitations such as subjectivity in interpretation and constraints in handling complex image features. This research paper proposes an integrated deep learning approach utilizing pre-trained models—VGG16, ResNet50, and InceptionV3—combined within a unified framework to improve diagnostic accuracy in medical imaging. The method focuses on lung cancer detection using images resized and converted to a uniform format to optimize performance and ensure consistency across datasets. Our proposed model leverages the strengths of each pre-trained network, achieving a high degree of feature extraction and robustness by freezing the early convolutional layers and fine-tuning the deeper layers. Additionally, techniques like SMOTE and Gaussian Blur are applied to address class imbalance, enhancing model training on underrepresented classes. The model’s performance was validated on the IQ-OTH/NCCD lung cancer dataset, which was collected from the Iraq-Oncology Teaching Hospital/National Center for Cancer Diseases over a period of three months in fall 2019. The proposed model achieved an accuracy of 98.18%, with precision and recall rates notably high across all classes. This improvement highlights the potential of integrated deep learning systems in medical diagnostics, providing a more accurate, reliable, and efficient means of disease detection.

## Introduction

Medical imaging is a pivotal tool for diagnosing a plethora of diseases, with lung cancer being one of the most critical due to its high mortality rate worldwide. Lung cancer, characterized by the uncontrolled growth of abnormal cells in one or both lungs, typically presents through various radiographic manifestations such as nodules, masses, or unusual opacities. The early detection and accurate classification of these manifestations into benign (non-cancerous) or malignant (cancerous) categories are crucial for effective treatment planning and improved patient outcomes [1, 2].

**Fig. 1:**
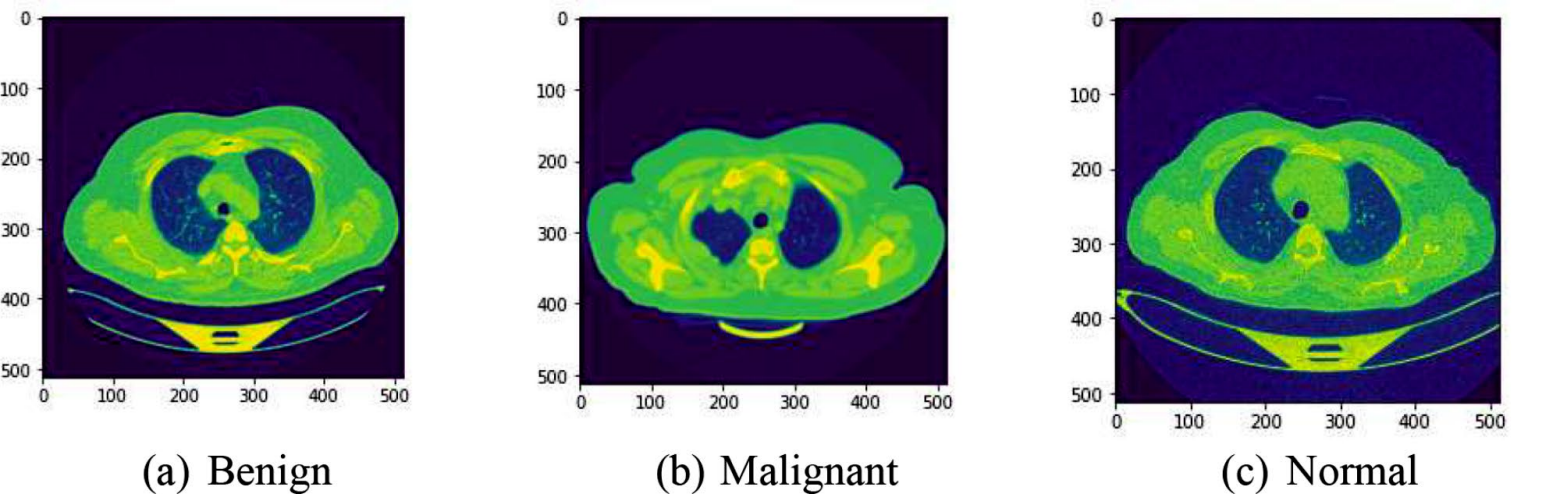
Sample images of different phases of lung cancer. **(a)** Benign. **(b)** Malignant. **(c)** Normal

Existing automated models primarily use basic machine learning algorithms that lack the depth necessary to understand complex image patterns fully. These models are also hindered by significant issues such as imbalanced datasets where certain disease manifestations are underrepresented, leading to biased predictions and reduced accuracy. Moreover, many of these systems do not effectively integrate the advancements in deep learning that have been proven to enhance feature extraction and classification tasks [3]. In Fig. 1 sample images from the dataset has been shown for better visual insights.

This study proposes an integrated deep learning approach that harnesses the capabilities of three state-of-the-art pre-trained models: VGG16, ResNet50, and InceptionV3. By utilizing a combination of these models, our approach aims to extract a richer set of features from medical images, which are crucial for accurate disease detection. The integration of multiple models is expected to leverage the unique strengths of each architecture, thereby providing a more robust analysis than could be achieved by any single model. Furthermore, the application of the Synthetic Minority Over-sampling Technique (SMOTE) addresses the issue of class imbalance, enhancing the model’s training process and its sensitivity towards less frequent conditions. Table 1 provides insights of different types of lung tissue and their characteristics.

**Table 1:** Characteristics of different types of tissues

Characteristic	Benign lung cancer	Malignant lung cancer	Normal lung tissue
Growth pattern	Slow, controlled growth	Rapid, uncontrolled growth	Balanced, regulated growth
Cell characteristics	Resemble normal cells	Abnormal, often with pleomorphic features	Normal appearance
Metastasis	Rarely metastasizes	Likely to metastasize to other organs	Does not metastasize
Prognosis	Generally good, less likely to be life-threatening	Poor, can be life-threatening	Normal, no cancer present
Treatment	Often requires monitoring, may not require aggressive treatment	Typically requires aggressive treatment (surgery, chemotherapy, radiation)	No treatment needed

### Motivation of the study

The research outlined in this manuscript addresses critical challenges in medical diagnostics, specifically enhancing the accuracy and reliability of lung cancer detection through imaging using deep learning. Integrating multiple pre-trained networks such as VGG16, ResNet50, and InceptionV3 presents technical challenges in harmonizing outputs and maintaining model stability. This raises the question of how to combine these architectures to enhance feature extraction without compromising generalizability. Additionally, handling class imbalances in medical datasets is essential to ensure fair representation and accuracy across all classes, prompting an investigation into the most effective methods for this. The adaptability of deep learning models to different types of medical imaging data is another challenge, as models need to maintain high accuracy and reliability across varying formats and resolutions. The usability and interpretability of these models in clinical settings are also crucial for assisting radiologists and improving clinical workflows, necessitating strategies to enhance these aspects. Finally, rigorous validation and verification processes are required to ensure that the models perform consistently across various datasets and real-world conditions, ensuring robustness, reliability, and safety for clinical use. Addressing these research questions is motivated by the potential of deep learning to significantly improve the accuracy, efficiency, and reliability of lung cancer diagnostics, ultimately leading to earlier detection and more effective treatment, and thereby pushing the boundaries of current technologies to create a standardized, objective, and reliable diagnostic tool.

### Objective of the research

The objectives of this research paper are summarized as follows:

- To construct a model using VGG16, ResNet50, and InceptionV3 to improve feature extraction for lung cancer image classification.
- To achieve superior accuracy, precision, and recall in classifying lung cancer images to improve diagnostic reliability along with interpretability through techniques like grad-cam.
- To implement techniques like SMOTE and Gaussian Blur to balance class distribution in medical imaging datasets.
- To design the model for ease of use and interpretability in clinical settings to support radiologists and enhance diagnostic workflows.

The remainder of the paper is organized as follows: Section II reviews related work in the field of medical image analysis, highlighting previous approaches and their limitations. Section III describes the methodology, including the dataset used, the preprocessing steps undertaken, the architecture of the integrated model, and the training process. Section IV presents the results of our experiments, including performance metrics such as accuracy, precision, and recall. Section V discusses the implications of our findings, the advantages of our approach over existing methods, and potential areas for future research. Finally, Section VI concludes the paper by summarizing the key outcomes and the impact of this study on the field of medical diagnostics.

## Related work

The analysis of lung cancer through medical imaging has been an area of extensive research, where diverse methodologies have been explored to enhance the accuracy and efficiency of diagnoses. This section reviews several prominent approaches employed in the automated analysis of lung cancer images, detailing the techniques and their respective advantages and limitations.

Initially, traditional machine learning algorithms such as Support Vector Machines (SVM), Decision Trees, and k-Nearest Neighbors (k-NN) were widely used. These methods often required extensive feature engineering, where domain experts manually identified and extracted relevant features from the images before classification [4]. While these methods were somewhat effective, they were heavily reliant on the quality of feature extraction and suffered from poor generalizability when faced with data from different imaging sources or patient demographics [5, 6]. With the advent of deep learning, Convolutional Neural Networks (CNNs) have become the cornerstone for medical image analysis, including lung cancer detection [7]. CNNs automatically learn to identify relevant features without the need for manual extraction, providing a significant leap in performance and adaptability [8].

More recently, transfer learning has gained traction where pre-trained models developed for general image recognition tasks are fine-tuned for specific medical imaging applications. This approach utilizes the learned features from vast and varied datasets like ImageNet to improve learning efficiency and accuracy in medical image analysis [9]. Ensemble methods that combine the predictions of multiple models to improve accuracy and robustness have also been explored [10]. Advanced methods like Synthetic Minority Over-sampling Technique (SMOTE) have been integrated into the training process to synthetically augment the minority classes by generating plausible examples. This approach helps in balancing the dataset, allowing models to learn more generalized features across all classes [11]. Table 2 presents several notable studies in the field of lung cancer classification.

**Table 2:** Studies on lung cancer classification

Study	Summary	Remarks
Shah, Asghar Ali et al. (2023) [12]	Deep learning ensemble 2D CNN for lung nodule detection achieves 95% accuracy, surpassing baseline methods.	Ensemble CNNs enhance lung nodule detection accuracy, showcasing deep learning’s potential in medical imaging.
Mikhael, Peter G. et al. (2023) [13]	Sybil model predicts individual lung cancer risk from LDCT scans with high accuracy, aiding in personalized screening.	Sybil facilitates personalized lung cancer screening, utilizing LDCT scans for early risk prediction.
Wankhade, Shalini & Vigneshwari (2023) [14]	CCDC-HNN combines deep learning and 3D-CNN for accurate early lung cancer diagnosis from CT scans, distinguishing benign and malignant tumors.	CCDC-HNN enhances early lung cancer detection, utilizing advanced deep learning techniques for improved accuracy.
Said, Yahia et al. (2023) [15]	Proposed system combines UNETR and self-supervised networks for accurate lung cancer diagnosis from CT scans, achieving high segmentation and classification rates.	Proposed system offers robust solution for early lung cancer diagnosis, leveraging advanced networks for improved accuracy.
Wani, Niyaz Ahmad et al. (2024) [16]	“DeepXplainer” hybrid model combines CNN and XGBoost for lung cancer detection, achieving high accuracy and interpretability.	“DeepXplainer” offers accurate lung cancer detection with transparent explanations, aiding in informed decision-making.
Chae, Kum Ju et al. (2023) [17]	Deep learning-based texture analysis detects interstitial lung abnormalities in CT scans, offering potential for improved assessment and management.	Deep learning-based texture analysis enhances ILA detection, providing insights for clinical practice improvement.
Guan, Peiyuan et al. (2023) [18]	Automated framework for PET image analysis using differential activation filter and CNN achieves superior performance, promising applications in medical imaging.	Proposed framework offers comprehensive solution for PET image analysis, addressing challenges in screening and segmentation.
Mohamed, Tehnan IA et al. (2023) [19]	EOSA-CNN hybrid algorithm achieves high accuracy in lung cancer classification, demonstrating potential for improved diagnosis in CT images.	EOSA-CNN offers promising approach for accurate lung cancer diagnosis, utilizing hybrid metaheuristic and CNN algorithms.
Rajasekar, Vani et al. (2023) [20]	Deep learning models analyze histopathological slides for improved lung cancer detection, offering potential advancements in early diagnosis.	Deep learning models enhance lung cancer detection accuracy, particularly in analyzing histopathological images.
Deepapriya, B. S. et al. (2023) [21]	Deep learning techniques predict lung diseases from X-ray and CT images, aiming for effective early diagnosis.	Deep learning-based lung disease prediction offers potential for early diagnosis, aiding medical practitioners.

Furthermore, multiscale approaches that analyze images at various resolutions help capture both macroscopic and microscopic features, crucial for identifying lung cancer stages and types [22].

While significant progress has been made in the automated analysis of lung cancer images through advanced machine learning and deep learning techniques, challenges remain in terms of generalizability, efficiency, and integration into clinical workflows. The proposed study aims to address these issues by leveraging an integrated deep learning model that combines the strengths of multiple architectures and advanced techniques for handling class imbalance.

## Methodology

**Fig. 2:**
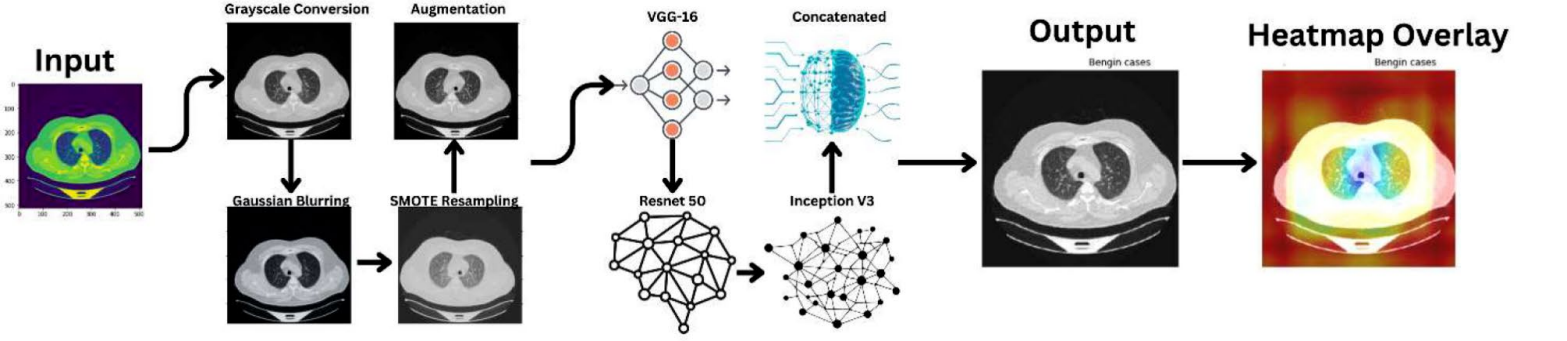
Proposed Model

The methodology adopted for this research focuses on a comprehensive approach to analyzing lung cancer images, addressing challenges in image variation and data imbalance. The process spans several stages, from data preprocessing and augmentation to advanced feature extraction and visualization techniques. Figure 2 demonstrates the proposed model’s workflow.

### Dataset description

The dataset utilized in this study comprises a collection of lung cancer images categorized into three distinct case types: Benign, Malignant, and Normal. These images were sourced from the IQ-OTHNCCD lung cancer dataset [23], a well-documented and publicly accessible resource. The images exhibit variations not only in labeling but also in their dimensions, which introduces a layer of complexity in automated analysis. The most common image size across the dataset is 512 × 512 pixels, although there are notable exceptions with sizes such as 512 × 623, 512 × 801, and a few others that deviate significantly from the norm, such as 404 × 511.

Table 3 represents the distribution of the dataset.

**Table 3:** Dataset distribution

Type	Number of Samples
Benign	120
Malignant	561
Normal	416

**Fig. 3:**
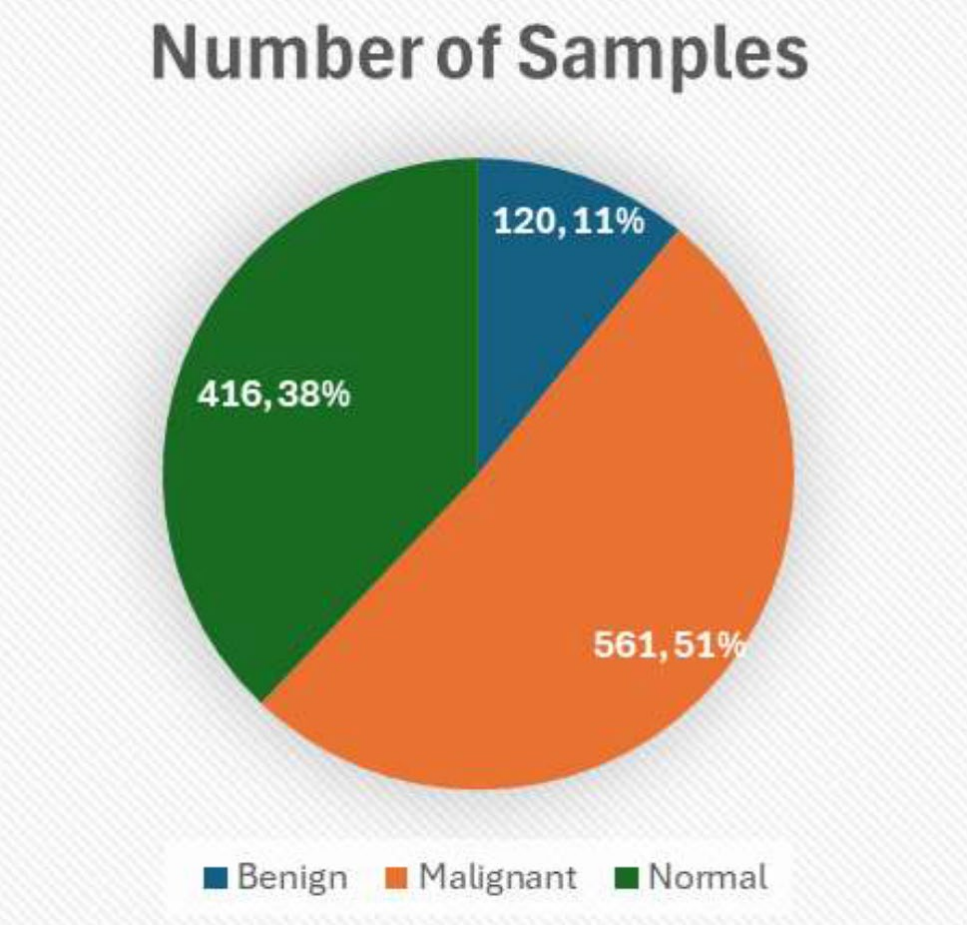
Distribution of dataset

Figure 3 demonstrated the above tabular data into visual form providing insights.

The dataset contains 120 Benign cases, all sized 512 × 512; Malignant cases are more varied with 501 images of size 512 × 512, along with 31 images of size 512 × 623, 28 images of size 512 × 801, and one image of size 404 × 511; Normal cases predominantly feature 415 images of size 512 × 512 with one outlier of size 331 × 506. This distribution is crucial for tailoring the preprocessing steps and ensures uniformity in input data for model training. This structured breakdown assists in the empirical analysis of the dataset and sets the foundation for subsequent image processing and analysis steps outlined in the methodology.

The IQ-OTH/NCCD lung cancer dataset, like many medical imaging datasets, is subject to potential biases that could affect the generalizability of any models trained on it. One significant concern is the dataset composition in terms of diversity—both in patient demographics (such as age, gender, and ethnicity) and in the range of medical imaging equipment used. If the dataset predominantly contains images from patients of a specific demographic or images captured using particular types of imaging technology, the model may not perform as well when exposed to data from broader, more diverse populations or different medical equipment. Additionally, the presence of class imbalance, where some classes (like benign, malignant, or normal cases) are underrepresented, could skew the model’s learning, leading it to overfit to the more frequently represented classes. This can result in poorer predictive performance on underrepresented classes, which is a critical issue in medical diagnostics where accuracy across all classes is vital. To mitigate these biases and enhance model robustness, it’s essential to use techniques such as data augmentation and advanced sampling methods like SMOTE for oversampling minority classes during training, and to validate the model across external datasets that are representative of the wider population.

### Dataset pre-processing

**Fig. 4:**
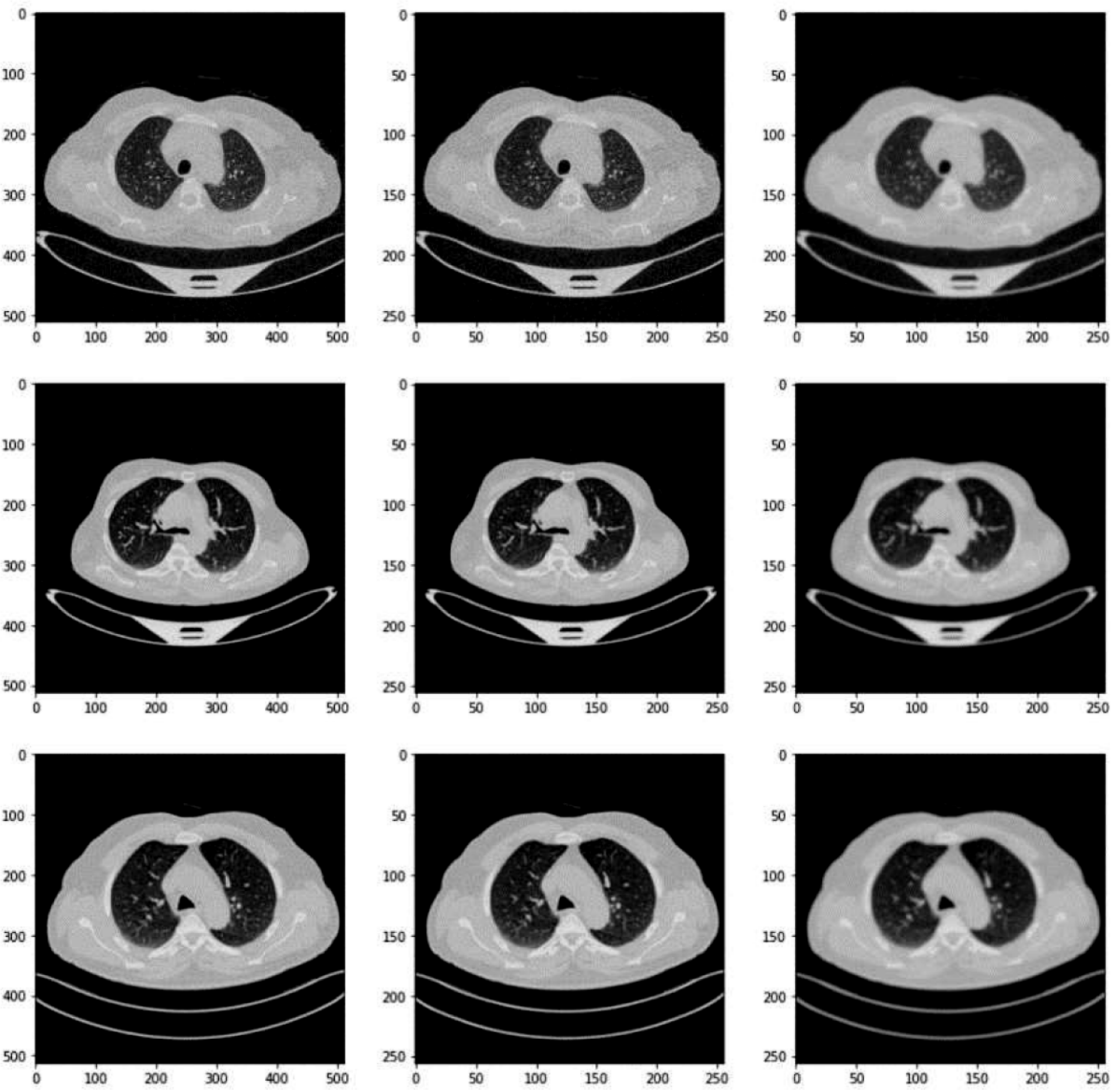
Basic pre-processed images

In the data preprocessing phase of our research, the initial step involved meticulously resizing each image within the lung cancer dataset to a standardized dimension of 256 × 256 pixels. This resizing is crucial as it ensures uniformity across all inputs, which is vital for consistent processing and analysis by the neural network models. The choice of 256 × 256 as a target size strikes a balance between retaining sufficient image detail for diagnostic purposes and reducing the computational load, thus enhancing the efficiency of the model training process. Some of the images after pre-processing have been shown in Fig. 4.

Following the resizing, we converted all images from their original RGB (red, green, and blue) color format to grayscale. Equation 1 resizes each original image to a standardized dimension of 256 × 256 pixels.

1 $${I}_{\text{resized}}=\text{resize}\left({I}_{\text{original}},256\times 256\right)$$

This conversion simplifies the input data by reducing it from three color channels to a single channel, focusing the model’s learning capacity on extracting relevant features from the textural and structural information present in the images, rather than color variations. Grayscale conversion is particularly beneficial in medical image analysis where color may not carry significant diagnostic information compared to texture and shape. Equation 2 converts resized images from RGB to grayscale format to simplify the input data.

2 $${I}_{\text{gray}}=\text{convert}\text{\_}\text{to}\text{\_}\text{gray}\left({I}_{\text{resized}}\right)$$

The final step in the preprocessing routine involved normalizing the pixel values of the grayscale images. Normalization is a critical process that scales down the pixel values to a range of 0 to 1. This is achieved by dividing each pixel value by 255, the maximum possible value in an 8-bit grayscale image. Equation 3 normalizes the pixel values of grayscale images to the range [0, 1] by dividing each pixel value by 255.

3 $${I}_{\text{normalized}}=\frac{{I}_{\text{gray}}}{255}$$

Normalizing the data to this range is a widely recognized best practice in machine learning as it ensures that all input features (pixel values, in this case) contribute equally to the learning process, preventing any single feature from dominating the model’s learning due to its scale. Furthermore, this normalization helps stabilize the neural network’s training phase by smoothing the landscape of the optimization function, thus facilitating quicker and more reliable convergence during the learning process. This comprehensive preprocessing approach not only aids in the homogenization of the input data but also significantly boosts the efficiency and effectiveness of the subsequent model training stages.

The preprocessing of the lung cancer dataset images is a crucial step in our methodology to ensure that the input data is uniform and suitable for effective model training. We resize all images to 256 × 256 pixels, a decision based on balancing computational efficiency with the preservation of essential diagnostic details. This uniform dimension allows our convolutional neural network (CNN) to process the images more efficiently and ensures consistency across all inputs, which is vital for the learning process. Additionally, each image undergoes grayscale conversion to reduce complexity and focus the model on textural and shape-related features rather than color, which is less relevant in this medical imaging context. The resizing is performed using OpenCV’s interpolation, which helps in preserving the quality of images during the size reduction. This standardization of image size and color simplifies the network architecture requirements and reduces the computational demand, crucial for the practical deployment of the model in medical diagnostics where resources may be limited.

### Data augmentation and data handling

In our study, we employed a comprehensive suite of data augmentation techniques aimed at enhancing the model’s robustness and mitigating the risk of overfitting, thereby ensuring better generalization across new and unseen data. The augmentation process included various transformations such as rotations, translations, horizontal flipping, and Gaussian blurring, each carefully chosen to mimic real-world variations encountered in medical imaging. Specifically, images were randomly rotated by angles between − 10 and 10 degrees to account for the different orientations that lung structures can assume during scanning. Equation 4 randomly rotates images by angles between − 10 and 10 degrees to simulate different orientations encountered in medical imaging.

4 $${I}_{\text{rotate}\text{d}}=\text{rotate}\left({I}_{\text{original}},{\theta }\right),\hspace{1em}-10\le {\theta }\le 10$$

We also applied translations, shifting images horizontally and vertically by up to 10% of the image size, which helps the model adapt to variations in lung positioning within the scanner field. Equation 5 applies translations to shift images horizontally and vertically by up to 10% of the image size to simulate variations in lung positioning.

5 $$\eqalign{{I_{{\rm{translated}}}} = & {\rm{translate}}\left( {{I_{{\rm{original}}}},\,dx,\,dy} \right) \cr & ,\,\left| {dx} \right|,\left| {dy} \right| \le 0.1 \times {\rm{image}}\_{\rm{size}} \cr}$$

Horizontal flipping was used to further augment the dataset by creating mirror images, representing the natural variability in how images might be presented or processed clinically. Equation 6 flips images horizontally to create mirror images, introducing natural variability in how images might be presented or processed clinically.

6 $${I}_{\text{flipped}}=\text{flip}\left({I}_{\text{original}}\right)$$

Additionally, Gaussian blurring was introduced as a technique to simulate the effect of slight focus variations that can occur in real diagnostic settings, where blurring can affect the clarity of structural boundaries within the images. This approach not only diversifies the training data but also conditions the model to effectively handle practical diagnostic challenges by learning from data that closely mimics the variability seen in actual clinical environments. Equation 7 applies Gaussian blurring to simulate slight focus variations encountered in real diagnostic settings, enhancing the model’s ability to handle practical diagnostic challenges.

In our image preprocessing pipeline, Gaussian Blur is critical for reducing noise and emphasizing relevant structures. We used a 5 × 5 kernel size, providing moderate blurring to smooth out noise without distorting essential lung tissue details. The blur intensity, or standard deviation, was set to zero, allowing automatic calculation based on the kernel size. This ensures optimal blurring tailored to the kernel size.

7 $${I}_{\text{blurred}}=\text{blur}\left({I}_{\text{original}}\right)$$

In terms of addressing the issue of imbalanced data, our initial examination of the dataset revealed a pronounced disparity in the distribution of classes, with ‘Malignant’ cases being substantially more prevalent than ‘Benign’ and ‘Normal’ cases. Such an imbalance can skew the model’s predictions towards the majority class. To counteract this, we employed the Synthetic Minority Over-sampling Technique (SMOTE), which is designed to balance the dataset by artificially synthesizing new examples in the minority classes. SMOTE works by identifying feature space similarities between existing examples in the minority class and generating new, synthetic samples that combine features of these close neighbors, effectively enriching the dataset with more diverse examples of underrepresented classes. Table 4 shows the before and after SMOTE data.

**Table 4:** Before and after SMOTE

Type	Before SMOTE	After SMOTE
Benign	420	420
Malignant	312	420
Normal	90	420

**Fig. 5:**
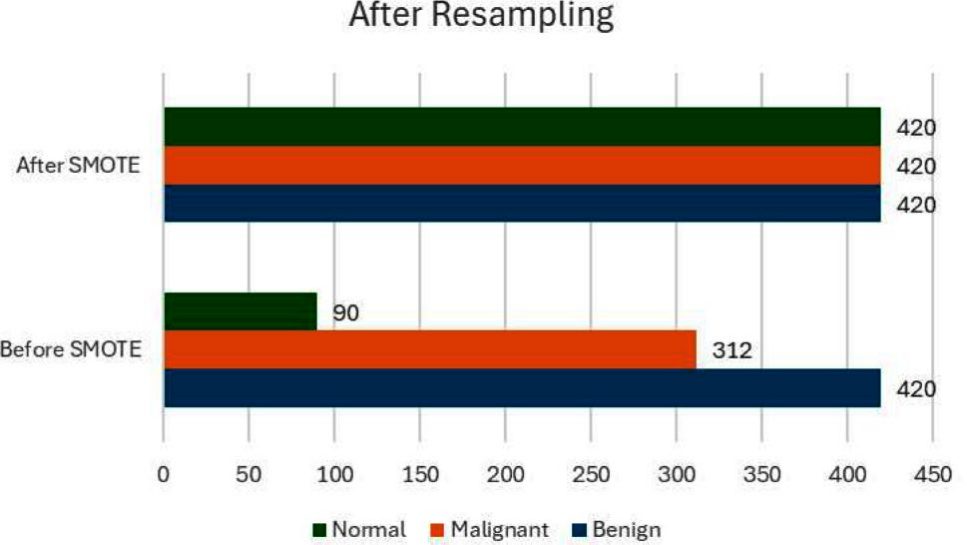
Before and after SMOTE

Figure 5 shows the analysis of the data before and after smote.

This ensures that all classes have equal representation in the training process, allowing the model to learn to recognize and differentiate features associated with all categories with the same level of accuracy, thereby reducing bias in the model’s predictions and enhancing its diagnostic reliability across all types of cases.

### Feature extraction and mapping

**Fig. 6:**
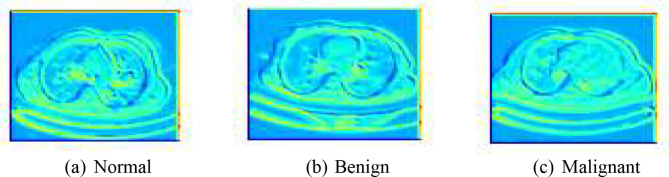
Feature extraction of the models. **(a)** Normal, **(b)** Benign, **(c)** Malignant

In our research, the process of feature mapping and extraction is crucial for enhancing the efficiency and accuracy of our predictive models. We utilized Principal Component Analysis (PCA) as a primary technique for dimensionality reduction and feature extraction. PCA assists in identifying the most relevant features from the large sets of image data by transforming the original data into a new set of variables, which are linear combinations of the original variables and are ordered so that the first few retain most of the variation present in all of the original variables. The decision on the number of components in PCA was strategically made based on the cumulative explained variance ratio, which guides us to choose a number of principal components that capture a substantial amount of information, while significantly reducing the dimensionality of the data. This approach not only simplifies the model but also speeds up subsequent training processes without sacrificing critical information. Figure 6 shows the feature extraction of the models.

Alongside PCA, we leveraged the power of pre-trained deep learning models—specifically VGG16, ResNet50, and InceptionV3—to extract deep features from the images. In our methodology, the VGG16, ResNet50, and InceptionV3 models were leveraged as the backbone for feature extraction, harnessing their powerful, pre-trained convolutional bases. Specifically, we employed these models up to their respective convolutional layers while keeping their initial weights intact to utilize the rich feature representations they have learned from extensive ImageNet datasets. For instance, for VGG16, we extracted features up to the fifth convolutional block (block5_conv3), which is known for capturing high-level features. Similarly, for ResNet50 and InceptionV3, we utilized outputs up to the activation layers just before their respective global pooling, ensuring a broad yet relevant spectrum of features are used. This approach allows the network to benefit from deep and complex architectures, ensuring robust feature extraction which is critical for the accuracy of classifying lung cancer images. By freezing these pre-trained layers, we significantly reduce the computational overhead during training, focusing the learning process on the new data-specific layers added atop the frozen architecture, which were fine-tuned to our specific lung cancer dataset.

### Model architecture

The composite model developed for this research integrates features from three state-of-the-art pre-trained convolutional neural networks (CNNs): VGG16, ResNet50, and InceptionV3. Each of these models has been extensively validated in the field of computer vision, particularly in tasks involving image classification and recognition. The choice to combine these networks stems from their unique architectural merits, which when combined, enhance the model’s feature extraction capabilities and robustness.

VGG16: Developed by Visual Graphics Group at Oxford, VGG16 is characterized by its simplicity, using only 3 × 3 convolutional layers stacked on top of each other in increasing depth. Reducing volume size is handled by max pooling. VGG16 is very effective in extracting low-level features from images but comes with a large number of trainable parameters, which makes it computationally intensive.

ResNet50: Short for Residual Network, ResNet50 utilizes skip connection, or shortcut connections, that allow it to skip one or more layers. The primary advantage of ResNet structures is their ability to enable very deep networks by addressing the vanishing gradient problem through these residual links. This allows the network to learn an identity function, ensuring that the higher layers will perform at least as good as the lower layers, and potentially better.

InceptionV3: This model is known for its efficiency in computing resources, utilizing a factorization concept into smaller convolutions. InceptionV3 layers apply multiple filters to an input and then concatenate the outputs. This setup allows the model to look at the same data in different ways, capturing cross-channel correlations and spatial correlations effectively.

**Fig. 7:**
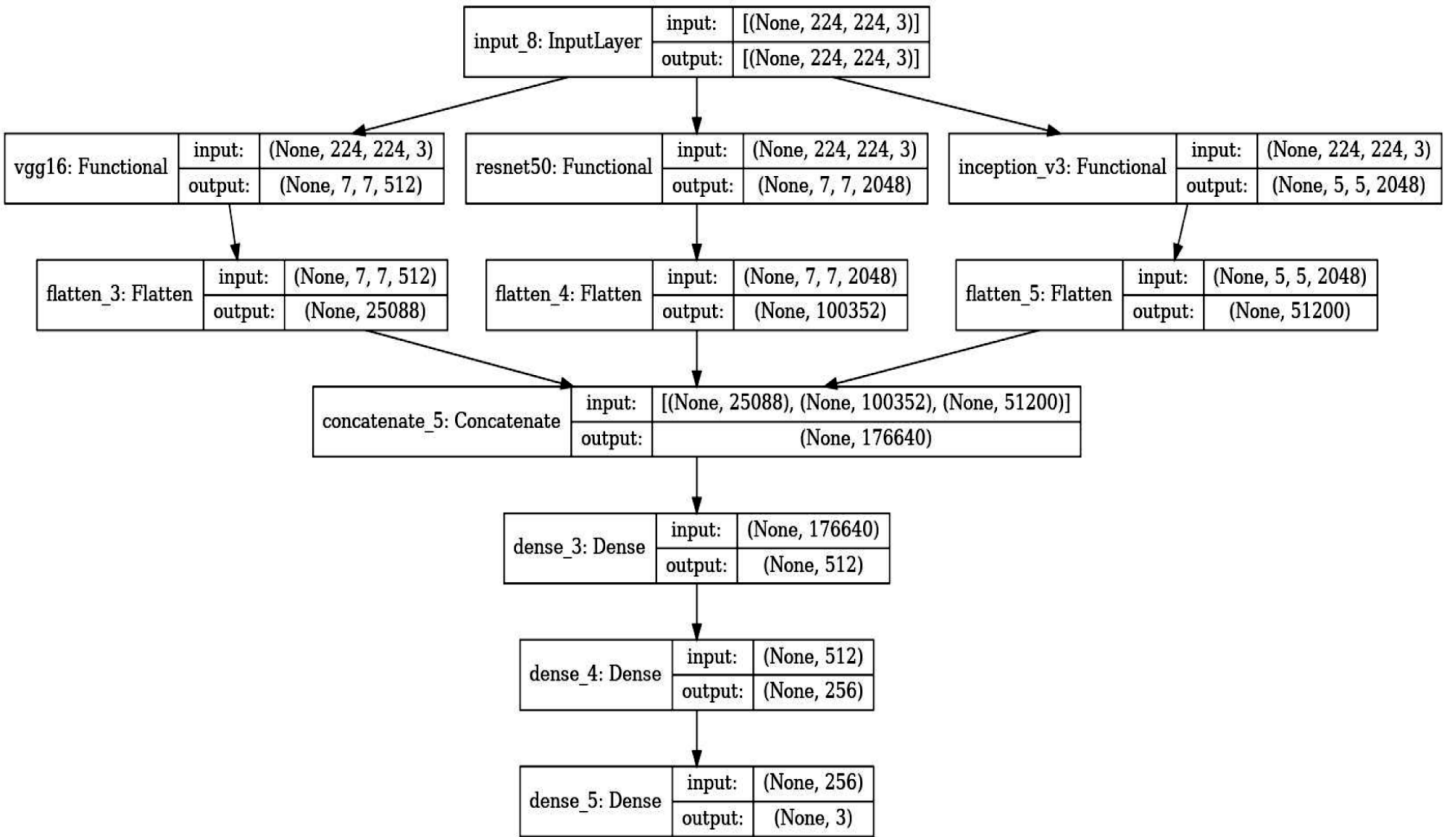
Architecture of the proposed model

In our model, each pre-trained network serves as a feature extractor where the final fully connected layers are removed, and the output feature maps are flattened and concatenated. This concatenated feature vector contains comprehensive information captured by different architectures. It feeds into a dense layer with a high degree of non-linearity to integrate these features effectively, followed by a final output layer with a softmax function for classification into three classes: Benign cases, Malignant cases, and Normal cases. The choice of using a softmax activation function in the final layer of our lung cancer detection model is pivotal for accurate classification and decision-making, especially in borderline cases. Softmax is ideal for our multi-class task—classifying images into Benign Cases, Malignant Cases, or Normal Cases—by converting raw predictions into probabilities. This transformation offers a clear probability distribution across classes, aiding in nuanced assessments where distinctions between classes are less clear. This probabilistic approach supports clinical decision-making by indicating the model’s confidence in each classification and allowing for cautious handling of uncertain cases through thresholding. It enhances interpretability by providing clinicians with transparent reasoning behind the model’s predictions, crucial for trust and adoption in medical settings. Table 5 shows the layers and description of the proposed model. The Fig. 7 shows the architecture of the proposed model in detail.

**Table 5:** Layers of the proposed model

Layer	Description
input_4	Input layer for images
vgg16	VGG16 model output
resnet50	ResNet50 model output
inception_v3	InceptionV3 model output
flatten	Flatten layer for VGG16 model output
flatten_1	Flatten layer for ResNet50 model output
flatten_2	Flatten layer for InceptionV3 model output
concatenate_2	Concatenation of flattened features from all three models
dense	Dense layer with 4096 neurons
dense_1	Dense layer with 4096 neurons
dense_2	Output layer with 3 neurons for classification (Benign, Malignant, Normal)

The extracted features from each of the pre-trained models are flattened and concatenated to form a single feature vector. This concatenated vector forms the input to a dense layer followed by the final classification layer. The rationale behind using a concatenated model lies in its ability to leverage diverse feature representations from multiple architectures, thereby enhancing the model’s ability to generalize across different visual representations of lung cancer cases.

The final layer of the model is a dense layer with softmax activation function that classifies an image into one of three categories: Benign cases, Malignant cases, or Normal cases. The softmax function is used because it outputs the probability distribution over the three classes, which is useful for classification.

Before training, all images were resized to match the input size requirements of the largest model (InceptionV3 requires 299 × 299 pixels, whereas VGG16 and ResNet50 require 224 × 224 pixels). Data augmentation techniques such as random rotations, shifts, zoom, and horizontal flipping were applied to create a robust model less prone to overfitting. Algorithm 1details about the methodology used in the proposed study.

**Algorithm 1:** Algorithm of proposed model

Input: Image for Classification Output: Classified into Benign, Malignant and Normal
**Step 1: Data Preparation**
1. Load dataset containing lung images categorized into Benign, Malignant, and Normal cases.
2. Preprocess images: Resize to 299 × 299 (InceptionV3), convert to grayscale if needed, and normalize pixel values.
3. Augment data: Apply rotation, shifting, zoom, flipping, SMOTE, and Gaussian Blurring.
**Step 2: Feature Extraction with Pre-trained Models**
1. Load Pre-trained Models: VGG16, ResNet50, InceptionV3.
2. Extract Features: Pass preprocessed images through models, flatten outputs.
**Step 3: Feature Concatenation and Model Architecture**
1. Concatenate Features: Combine flattened features from all models.
2. Build Composite Model: Add dense layer (512 neurons, ReLU), dropout (0.5), and output layer (softmax).
**Step 4: Model Compilation**
1. Compile Model: Loss - sparse categorical crossentropy, Optimizer - Adam (lr = 0.001), Metrics - accuracy, precision, recall, F1-score.
**Step 5: Model Training**
1. Split Data: Training, validation, test sets.
2. Train Model: Define batch size, epochs. Use callbacks (Early Stopping, LR Scheduler).

The composite model was compiled using the Adam optimizer, which adjusts the learning rate throughout training, and sparse categorical cross entropy as the loss function, ideal for multi-class classification of mutually exclusive classes. We chose the Adam optimizer for its robust performance in handling complex medical image datasets. Adam adapts learning rates for each parameter based on gradient estimates, making it efficient for sparse and noisy data typical in medical imaging. It offers advantages such as adaptive learning rates, computational efficiency, and robustness to gradient scaling. While Adam excels in initial convergence, comparing it with alternatives like SGD, RMSprop, and Nesterov Accelerated Gradient can reveal optimal choices for specific operational needs, such as generalization and stability on unseen data. This evaluation ensures our model is finely tuned for clinical application, balancing performance and efficiency in training. Table 6 shows the training parameters.

**Table 6:** Training parameters

Parameters	Value
Batch Size	8
Epochs	20
Learning Rate	0.001
Decay	0.1

Dropout layers with a rate of 50% were interspersed between dense layers to reduce overfitting by randomly deactivating certain neurons during training.

**Fig. 8:**
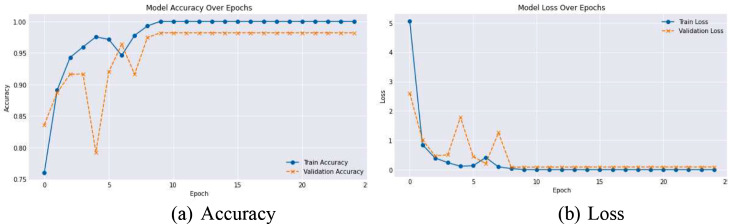
Accuracy and Loss over Epochs. **(a)** Accuracy, **(b)** Loss

Alongside monitoring the accuracy during training, validation was rigorously performed using metrics such as precision, recall, and F1-score to understand the model’s performance across different classes, providing insights into any class imbalances handling. Figure 8 shows the training and validation loss of the proposed model.

Equation 8 represents loss function measures the difference between the true distribution *y* and the predicted distribution ​$\widehat{y}$ for multi-class classification tasks where the classes are mutually exclusive.

8 $$L\left(y,\widehat{y}\right)=-{\sum }_{i=1}^{n}{y}_{i}\text{log}\left(\widehat{{y}_{i}}\right)$$

Where,

- 𝑦_𝑖_​ represents the true probability distribution of the 𝑖th class.
- $\widehat{y}$ represents the predicted probability that the input belongs to the 𝑖th class.

Equation 9 to 11 represents the adam which is an adaptive learning rate optimization algorithm that computes adaptive learning rates for each parameter. It combines the advantages of AdaGrad and RMSProp algorithms.

9 $$m\leftarrow {{\beta }}_{1}m+\left(1-{{\beta }}_{1}\right)g$$

10 $$v\leftarrow {{\beta }}_{2}v+\left(1-{{\beta }}_{2}\right){g}^{2}$$

11 $$\theta \leftarrow \theta - {\alpha \over {\sqrt v + \in }}m$$

where:

- 𝑚 and *v* are exponentially moving averages of the gradients and the squared gradients respectively.
- 𝑔 is the gradient of the objective function with respect to the parameters.
- 𝜃 represents the model parameters.
- 𝛼 is the learning rate.
- 𝛽_1_​ and 𝛽_2_​ are exponential decay rates for the moment estimates.
- 𝜖 is a small constant to prevent division by zero.

Equation 12 presents the Softmax function that converts the raw scores (logits) of each class into probabilities. It ensures that the sum of the probabilities of all classes is equal to 1, making it suitable for multi-class classification tasks.

12 $$\text{softmax}{\left(x\right)}_{i}=\frac{{e}^{{x}_{i}}}{{\sum }_{j}{e}^{{x}_{j}}}$$

Where,

- *x*_*i*_​ is the raw score (logit) for the 𝑖th class.

This detailed methodology ensures a deep understanding and harnessing of each model’s strengths, leading to a robust and highly accurate system for classifying lung cancer images.

### Model evaluation metrics

The evaluation of model performance is critical to understanding its effectiveness and usability in practical scenarios. This section outlines various metrics used to assess the model developed for classifying lung cancer images into benign, malignant, and normal categories. These metrics provide a comprehensive understanding of the model’s accuracy, reliability, and diagnostic ability.

**Accuracy** measures the overall correctness of the model and is defined as the ratio of correctly predicted observations to the total observations. It provides a quick indication of performance, especially in balanced datasets [24]. It is given in Eq. 13.

13 $$\text{Accuracy}=\frac{\text{True Positives}+\text{True Negatives}}{\text{Total Observations}}$$

**Precision** (Positive Predictive Value) measures the accuracy of positive predictions. It is defined as the ratio of true positive predictions to the total predicted positives. High precision relates to a low rate of false positives [25]. It is given in Eq. 14.

14 $$\text{Precision}=\frac{\text{True Positives}}{\text{True Positives}+\text{False Positives}}$$

**Recall** (Sensitivity) indicates the ability of the model to find all relevant cases within a dataset. It is defined as the ratio of true positives to the actual number of positives. High recall relates to a low rate of false negatives. It is given in Eq. 15.

15 $$\text{Recall}=\frac{\text{True Positives}}{\text{True Positives}+\text{False Negatives}}$$

The **F1-score** is the harmonic mean of precision and recall. It is particularly useful when the class distribution is uneven. The score takes both false positives and false negatives into account and is a better measure of the incorrectly classified cases than the Accuracy Metric. It is given in Eq. 16.

16 $$\text{Recall}=\frac{\text{True Positives}}{\text{True Positives}+\text{False Negatives}}$$

The **ROC curve** is a graphical plot that illustrates the diagnostic ability of a binary classifier system as its discrimination threshold is varied. The **AUC** represents a degree of separability. It tells how much the model is capable of distinguishing between classes. Higher the AUC, better the model is at predicting 0s as 0s and 1s as 1s [26]. It is given in Eq. 17.

17 $$\text{AUC}={\int }_{0}^{1}\text{TPR}\left({\text{FPR}}^{-1}\right)d\left({\text{FPR}}^{-1}\right)$$

A **confusion matrix** is a table that is often used to describe the performance of a classification model on a set of test data for which the true values are known. It allows visualization of the performance of an algorithm. Each row of the matrix represents the instances in a predicted class, while each column represents the instances in an actual class (or vice versa) [27].

**Precision-Recall Curve** curve plots the precision (y-axis) and the recall (x-axis) for different probability thresholds. It helps in identifying the trade-off between recall and precision for different thresholds. A higher area under the curve represents both high recall and high precision.

**Cohen’s Kappa** is used to measure inter-rater reliability (and also intra-rater reliability) for qualitative (categorical) items. It is generally thought to be a more robust measure than simple percent agreement calculation since Kappa takes into account the agreement occurring by chance. Cohen’s Kappa is a better measure when you are dealing with imbalanced classes. It is given by Eq. 18.

18 $${\kappa }=\frac{P\left(A\right)-P\left(E\right)}{1-P\left(E\right)}$$

**Matthews Correlation Coefficient (MCC)** is used in machine learning as a measure of the quality of binary classifications. It takes into account true and false positives and negatives and is generally regarded as a balanced measure which can be used even if the classes are of very different sizes. It is given by Eq. 19.

19 $$\text{MCC}=\frac{\text{TP}\times \text{TN}-\text{FP}\times \text{FN}}{\sqrt{\left(\text{TP}+\text{FP}\right)\left(\text{TP}+\text{FN}\right)\left(\text{TN}+\text{FP}\right)\left(\text{TN}+\text{FN}\right)}}$$

**F2 score** weighs recall higher than precision (by placing more emphasis on false negatives). It is a measure of a test’s accuracy. It considers both the precision and the recall to compute the score. The F2 score can be particularly useful when you are more concerned about minimizing false negatives than false negatives. It is given by Eq. 20.

20 $${F}_{{\beta }}=\left(1+{{\beta }}^{2}\right)\cdot \frac{\text{Precision}\times \text{Recall}}{{{\beta }}^{2}\cdot \text{Precision}+\text{Recall}}$$

Incorporating these metrics provides a robust analysis of the model’s performance across various dimensions, essential for validating the effectiveness of the predictive model in a clinical setting. Each metric is computed using the validation data set to ensure the model’s generalizability to new, unseen data.

### Advanced visualization techniques

To enhance the interpretability of the deep learning models used for lung cancer image classification, advanced visualization techniques such as Gradient-weighted Class Activation Mapping (Grad-CAM) and Feature Map Visualization are employed. These techniques help in understanding what the model sees and which parts of the image are being focused on to make the predictions.

**Grad-CAM** (Gradient-weighted Class Activation Mapping) provides insights into which areas of the input image influenced the model’s decision. This method uses the gradients of any target concept (output of the model for a given class), flowing into the final convolutional layer to produce a coarse localization map highlighting important regions in the image for predicting the concept. The final convolutional layer is chosen because it captures high-level features in the image that are crucial for making predictions. Using TensorFlow’s GradientTape, the gradients of the target class (decided based on the model’s prediction) with respect to the output feature map of the selected layer are computed. These gradients indicate how much each neuron’s activity should change to affect the output class score. These gradients are pooled (using global average pooling) to obtain the neuron importance weights. The feature maps are then weighted by these importance values. The weighted feature maps are summed along the channel dimension and followed by a ReLU function to obtain a heatmap. This heatmap is then resized to the dimensions of the input image to show the focus areas. The heatmap is superimposed on the original grayscale image to visualize the areas most relevant to the model’s prediction. This helps in understanding why the model predicts certain cases as benign, malignant, or normal.

**Feature Map Visualization** allows us to see the output of individual convolutional layers and understand what features the model is extracting at different stages of the network. This is particularly useful to check whether the model is learning relevant patterns from the images. Depending on the architecture, several layers can be chosen to visualize the feature maps. Typically, earlier layers capture basic features like edges, while deeper layers capture more complex features like textures or specific shapes relevant to lung cancer patterns. The model is run forward with an input image up until the selected layers, and the outputs (feature maps) of these layers are extracted. Each feature map is visualized as an individual image. In practice, due to a large number of feature maps, only a subset may be visualized. For example, the first few feature maps might be displayed to show the variety of features detected by the layer. By analyzing these feature maps, researchers can determine if the model is focusing on meaningful information in the images (like tumors or irregular growths) or if it is being distracted by noise and irrelevant information.

Together, Grad-CAM and Feature Map Visualization provide powerful tools for understanding and debugging deep learning models, ensuring that the models are indeed learning to identify meaningful patterns in medical images rather than being influenced by confounding factors.

## Results

In this study, the development of a composite model utilizing features extracted from VGG16, ResNet50, and InceptionV3 has demonstrated outstanding performance in the classification of lung cancer images into Benign, Malignant, and Normal categories. This model achieved an overall accuracy of 98.18%, which underscores its effectiveness in clinical diagnostics, particularly in distinguishing subtle nuances between different types of lung conditions.

The model displayed exceptionally high precision and recall across all categories. Specifically, it achieved perfect precision for Malignant cases (1.0000) and nearly perfect recall (0.9929) for the same. For Benign cases, both precision and recall were 0.9333, and for Normal cases, the model scored 0.9714 in precision and 0.9808 in recall. These results highlight the model’s capability to correctly identify positive cases as such (precision) and its effectiveness in identifying all actual positive cases (recall). This balance is critical in medical imaging, where the cost of false negatives or false positives can be high. Table 7 presents the classification report.

**Table 7:** Classification report

Class	Precision	Recall	F1-Score
Benign (0)	0.9333	0.9333	0.9333
Malignant (1)	1.0000	0.9929	0.9964
Normal (2)	0.9714	0.9808	0.9761
Macro Avg	0.9683	0.9690	0.9686
Weighted Avg	0.9819	0.9818	0.9819

**Fig. 9:**
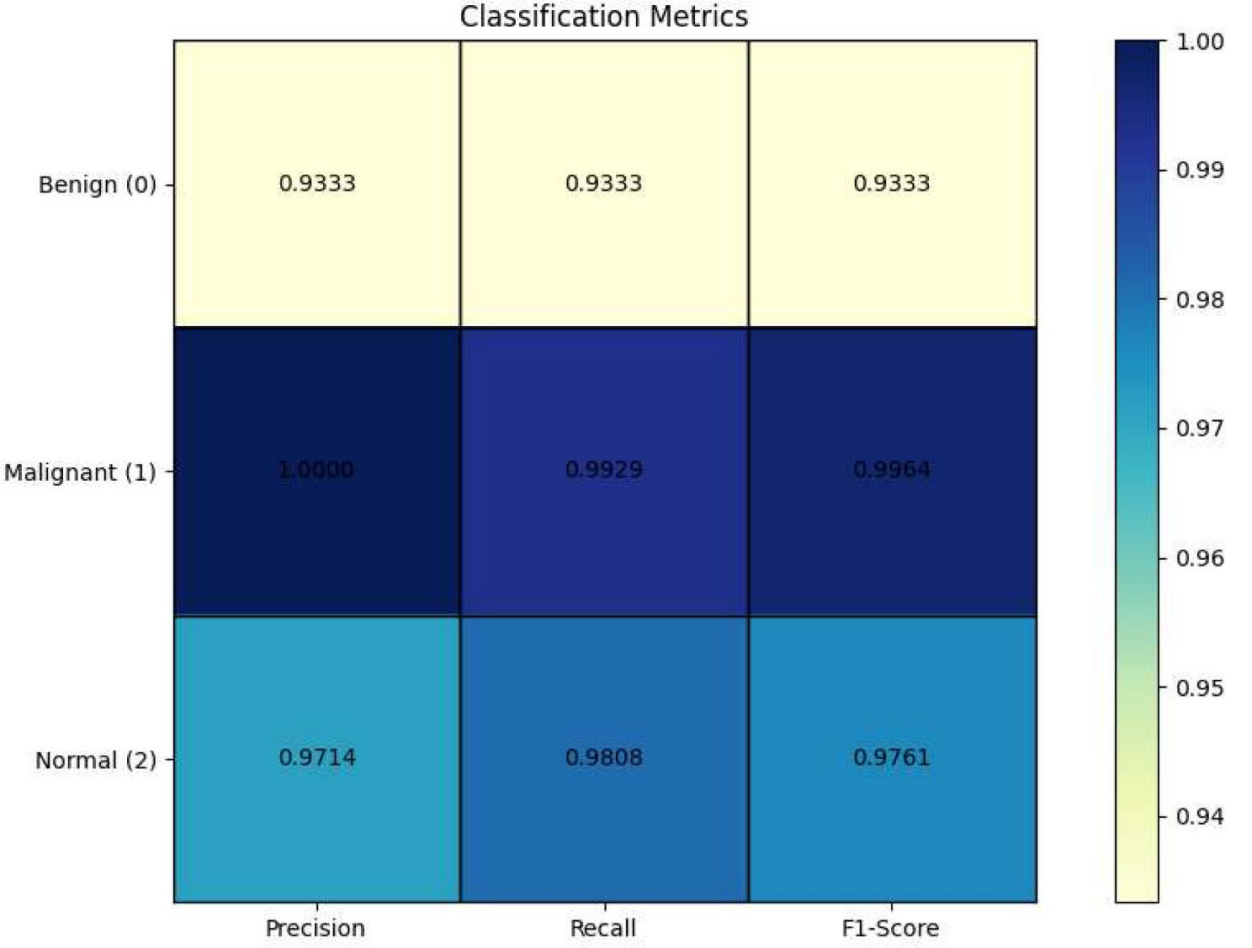
Classification report

The F1-score, which harmonizes the precision and recall, was notably high across the board, reinforcing the model’s balanced performance under various conditions. With scores such as 0.9333 for Benign, 0.9964 for Malignant, and 0.9761 for Normal, the model proves its consistent reliability and accuracy across diverse lung conditions. Figure 9 demonstrates the heatmap of the classification report.

The Matthews Correlation Coefficient (MCC) of 0.9688, almost at the perfect score, illustrates the quality of binary classifications performed by the model, indicating strong correlations between observed and predicted classifications. The Balanced Accuracy and Cohen’s Kappa Score, both around 0.969, signify the model’s uniform effectiveness across classes, particularly important in datasets where class distribution might not be uniform.

Error metrics provided further insights into the model’s performance: the Mean Squared Error (MSE) at 0.0618 and the Root Mean Squared Error (RMSE) at 0.2486 were particularly low, indicating that the model’s predictions were closely aligned with the actual data, with minimal average errors. The Mean Absolute Error (MAE) of 0.0327 highlighted that the model’s average predictions deviated only slightly from the true values, attesting to its precise predictive capabilities. Table 8 presents the advanced metrics and error metrics.

**Table 8:** Advanced and error metrics

Metric	Value
Matthews Correlation Coefficient	0.9688
Balanced Accuracy	0.9690
Cohen’s Kappa Score	0.9688
Mean Squared Error (MSE)	0.0618
Root Mean Squared Error (RMSE)	0.2486
Mean Absolute Error (MAE)	0.0327

**Fig. 10:**
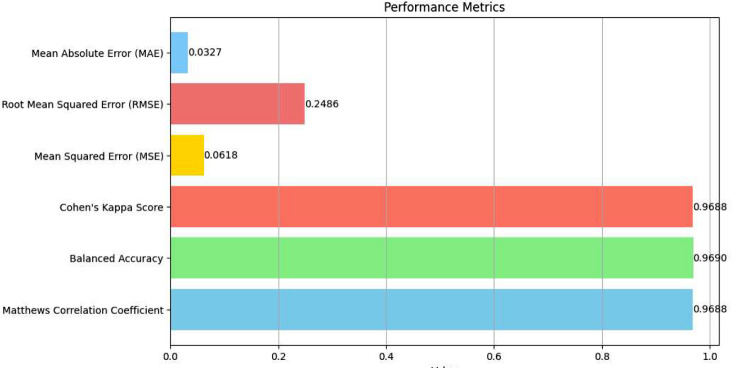
Advanced and error metrics

Figure 10 represents the visual of this metrics.

**Fig. 11:**
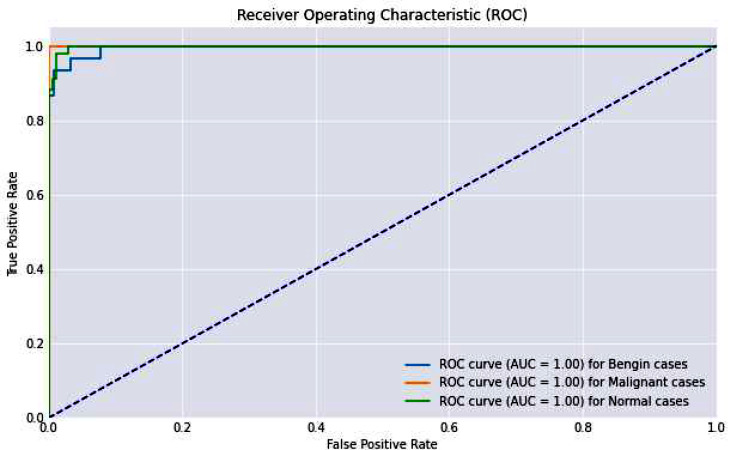
ROC-AUC Curve

The ROC-AUC and Precision-Recall curves, with near-perfect scores, effectively demonstrated the model’s diagnostic power. These curves are crucial in a clinical context as they provide a visual representation of the trade-off between sensitivity (true positive rate) and specificity (false positive rate), as well as between precision and recall. High values in these metrics reassure the model’s ability to serve as a reliable diagnostic tool. Figure 11 presents the roc-auc curve.

**Fig. 12:**
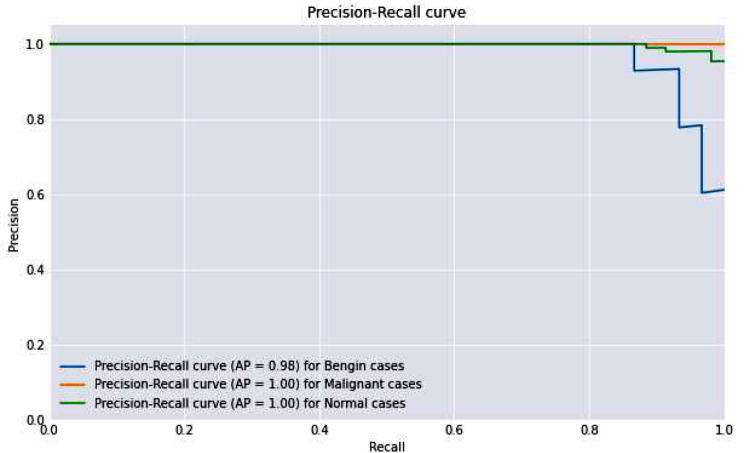
Precision and recall curve

Figure 12 presents the precision and recall curve of the proposed model.

**Fig. 13:**
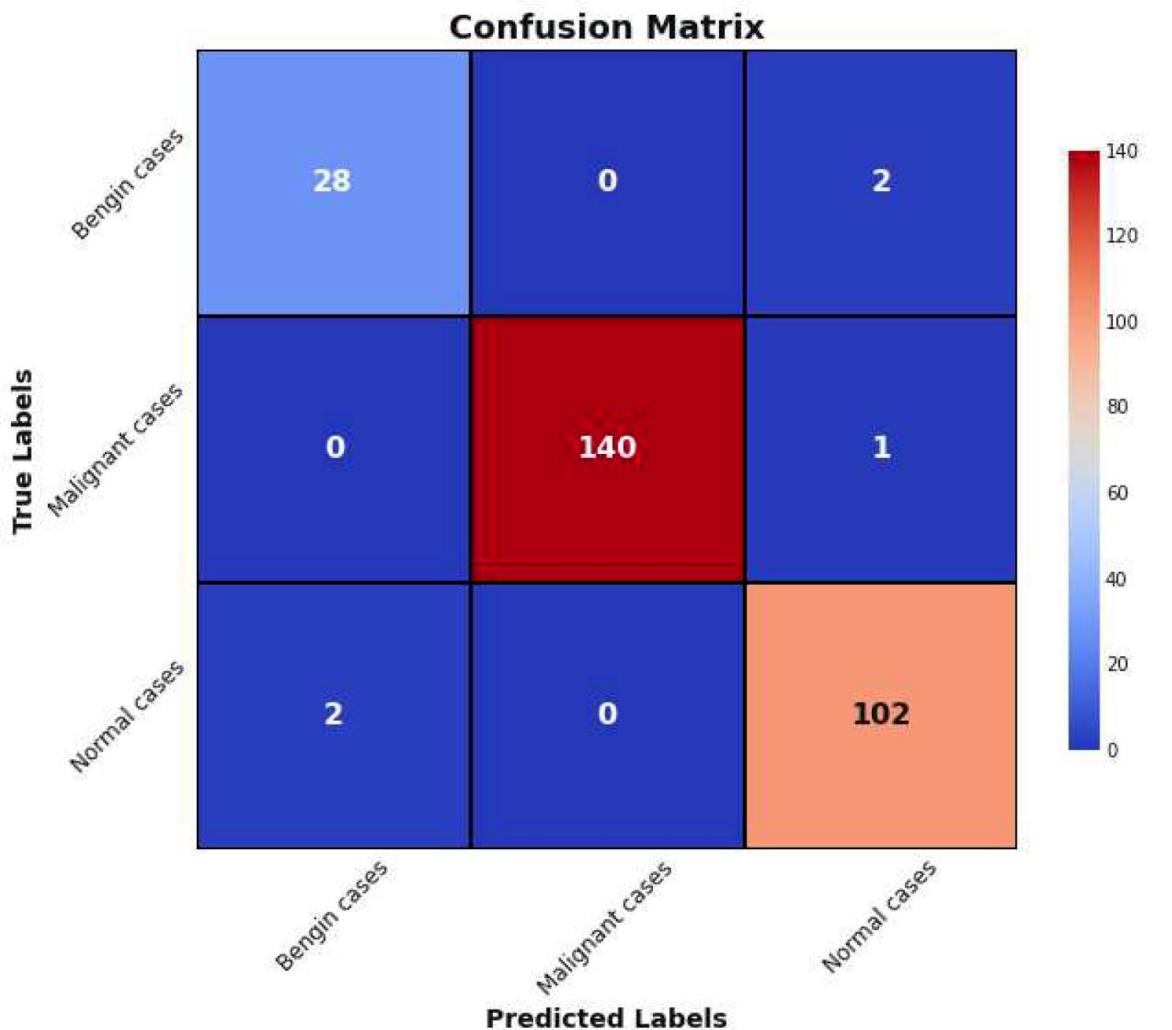
Confusion matrix

Figure 13 presents the confusion matrix for the proposed model.

The model achieved high accuracy in identifying Benign Cases, correctly classifying 28 out of 30 cases, with 2 cases misclassified as Normal Cases, highlighting a strong true positive rate but indicating a need for improvement in distinguishing benign from normal presentations, possibly due to overlapping features.

For Malignant Cases, the model performed exceptionally well, accurately classifying 140 out of 141 cases, with only one misclassification where a Malignant Case was predicted as Normal. This near-perfect detection underscores the model’s effectiveness in identifying distinct pathological features indicative of malignancy.

In Normal Cases, the model showed strong performance, correctly classifying 102 out of 104 cases, with 2 cases misclassified as Benign. This suggests challenges in fully excluding pathology in what are otherwise normal imaging findings, potentially due to benign features mimicking normal variations.

Types of Errors observed include minimal False Positives, mainly benign and malignant cases occasionally misclassified as normal, indicating conservative predictions of normalcy. False Negatives, however, are more prevalent, primarily benign and normal cases being misclassified as each other, posing clinical implications such as unnecessary procedures or missed follow-up.

**Fig. 14:**
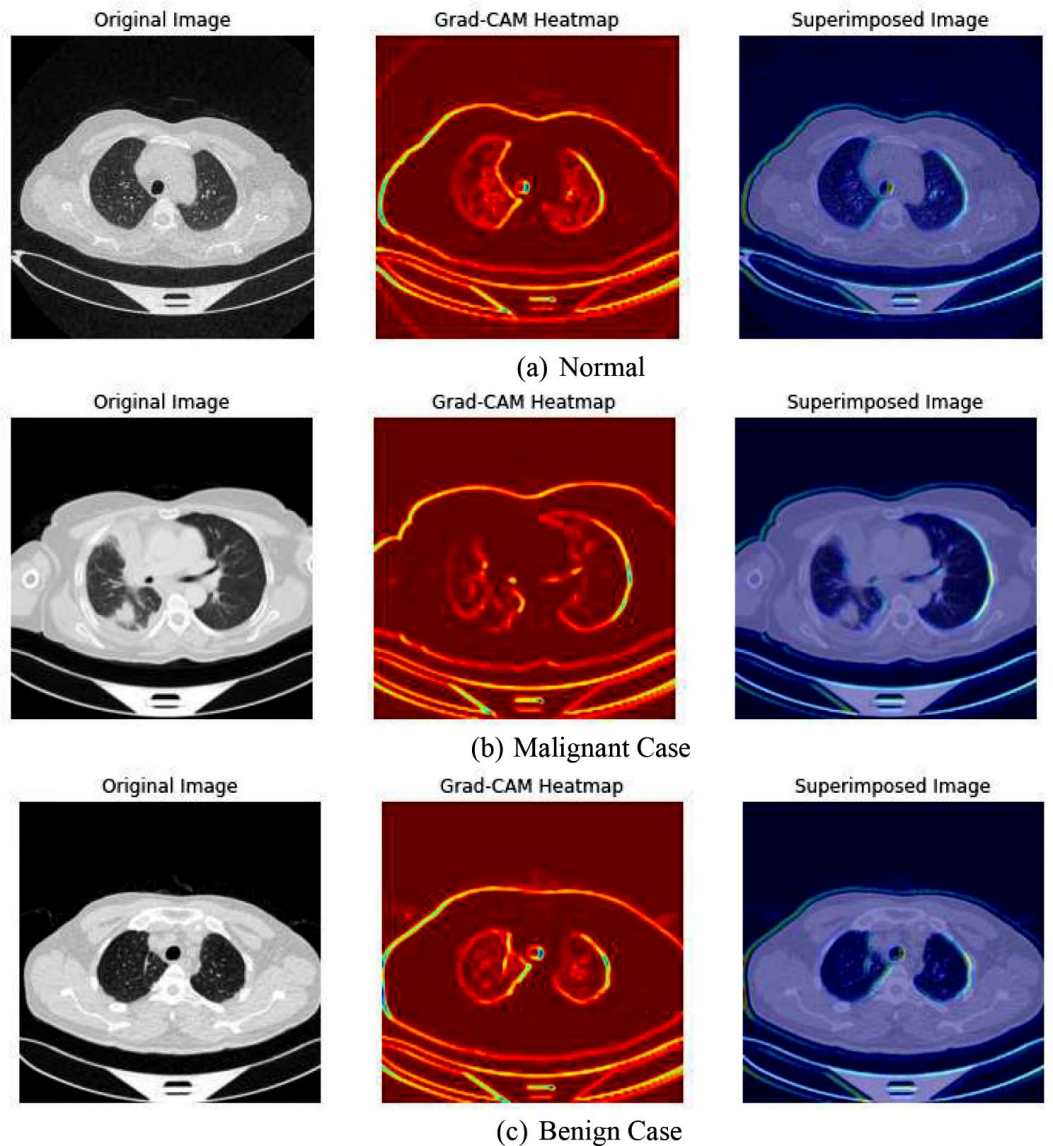
Grad-Cam and Superimposed Visualization. **(a)** Normal, **(b)** Malignant Case, **(c)** Benign Case

Finally, the application of Gradient-weighted Class Activation Mapping (Grad-CAM) provided a visual explanation of which areas in the images influenced the model’s predictions. These visualizations are invaluable as they allow clinicians to see which features in the lung scans are most indicative of specific classifications, adding an additional layer of interpretability to the model’s decision-making process. This can enhance trust in automated systems and assist in further diagnostic reasoning. Figure 14 represents the grad Cam visualization of the proposed model.

In our model, Grad-CAM is utilized to generate heatmaps that highlight influential regions in lung scans for classification. It captures gradients flowing into final convolutional layers relative to specific output classes like malignant or benign nodules. These gradients are pooled channel-wise to derive a localization map, emphasizing critical regions for class prediction. This process involves forward passing an image through the network, computing gradients with respect to feature maps, pooling gradients spatially, and combining weights with activation maps to produce heatmaps. Clinically, these heatmaps provide visual validation of the model’s focus on relevant areas, aiding in distinguishing pathologically significant features from potential artifacts or irrelevant regions. Grad-CAM enhances transparency and validation in AI-driven diagnostic assessments, supporting clinicians in making informed decisions.

Although it has several limitations that affect its utility in interpreting deep learning models. It primarily focuses on high-level features from later layers of the model, providing insights into major image features but lacking details on how mid or early-level features influence decisions. Spatial localization is constrained by the resolution of output feature maps, resulting in coarse heatmaps that may not accurately pinpoint small or subtle image features, crucial in clinical contexts. Additionally, Grad-CAM’s effectiveness varies with different neural network architectures, limiting its application across diverse models. The interpretative nature of heatmaps can lead to ambiguity, showing relevant areas without explaining their clinical significance or causal relationship. Over-reliance on Grad-CAM could foster false confidence in model interpretations, potentially misleading clinicians relying on these visualizations for decision-making.

This detailed performance breakdown showcases not only the model’s high accuracy but also its robustness and reliability across various metrics, making it an effective tool for enhancing diagnostic procedures in lung cancer detection.

## Discussion

The integration of convolutional neural networks (CNNs) such as VGG16, ResNet50, and InceptionV3 to form a composite model for lung cancer image classification has demonstrated notable success, achieving an accuracy of 98.18%. This achievement is significant, considering the critical nature of timely and accurate lung cancer diagnosis. The findings from this study illuminate several key advantages and implications that extend beyond the immediate results, pointing to broader impacts on the field of medical image analysis and potential pathways for future research.

The high precision and recall scores obtained by the composite model, particularly the perfect precision in classifying malignant cases, underscore its potential to serve as a reliable diagnostic aid. The ability of the model to correctly identify and classify lung cancer cases with minimal error could lead to earlier detection rates and better patient outcomes, especially in malignant cases where early intervention is crucial. Moreover, the application of advanced metrics like the Matthews Correlation Coefficient (MCC) and Balanced Accuracy not only confirms the model’s efficacy but also its consistency and reliability across varied cases, making it a robust tool for clinical use.

Compared to traditional diagnostic methods, which rely heavily on the expertise of radiologists and can be subjective, the use of a composite CNN model introduces a level of standardization and objectivity into the diagnostic process. One of the primary advantages of this approach is the reduction in human error and variability, which is often a challenge in medical imaging diagnostics. Additionally, existing single-model CNN approaches may not capture the full complexity of medical images due to their limited architectures. By integrating multiple pre-trained networks, our model leverages the strengths of each—VGG16’s texture sensitivity, ResNet50’s depth for feature extraction without gradient loss, and InceptionV3’s efficiency in processing different scales of image features. This integration allows for a more comprehensive analysis of lung images, enhancing the model’s ability to detect nuanced patterns indicative of various types of lung conditions. Table 9 presents the comparative study of the proposed model with the existing models.

**Table 9:** Comparative study

Author (Year)	Study Technique	Accuracy
Kusuma, S. (2024) [28]	Hybrid CNN-RNN Model with Pelican Optimization Algorithm	97.3%
Reshma, G. (2024) [29]	Deep Convolutional Neural Network (Deep CNN) and CNN	95%
Mohana Krishna, N. (2024) [30]	ResNet-50 and Inception V3 CNN Models	93.09%
Safta, Wiem (2024) [31]	Integration of 3D-LOP Descriptor, 3D-CNN, and Geometric Feature Analysis	97.84%
Mohmmad, Sallauddin (2024) [32]	Denoising Techniques with Classification using U-Net Architecture	97.15%
Pacal, Ishak (2024) [33]	Swin Transformer Architecture for Lung Cancer Detection	97.58%
Princy Magdaline, P. (2023) [34]	Attention Gate Residual U-Net Model, CNN, and KNN Classifier	97%
Ahnaf, Kern Cesarean (2023) [35]	GLCM and LBP Feature Extraction, SVM and Gaussian Naive Bayes	93%
Proposed Model	Integrated Deep Learning Approach utilizing Pre-trained Models, SMOTE, and Gaussian Blur	98.18

Moreover, the computational efficiency of using pre-trained models allows for quicker adaptation and implementation in clinical settings, where computational resources and time are often at a premium. The ability to use transfer learning also significantly reduces the amount of data required to train effective models, an important consideration given the difficulties in acquiring large annotated medical datasets.

To prevent overfitting and ensure our model’s reliability despite achieving high accuracy rates, we implemented several strategies. Data augmentation techniques such as rotations, flips, and zooms diversified the training set, enabling the model to learn general features effectively. Dropout layers were utilized to introduce redundancy by randomly deactivating neurons during training, promoting better generalization. Early stopping criteria were applied to halt training if validation accuracy plateaued, thereby preventing the model from overfitting to noise in the data. Cross-validation was employed to assess performance across different subsets of the dataset, ensuring robustness. Regular evaluation on a separate validation set further validated the model’s ability to generalize well to new data, affirming its practical reliability. Assessing computational costs for our model, which utilizes VGG16, ResNet50, and InceptionV3 architectures, is crucial for feasibility in clinical deployment. Training demands significant GPU resources, while efficient inference for real-time diagnostics is essential. Techniques like model quantization and pruning were explored to reduce model size without sacrificing performance, making it suitable for deployment on limited hardware. Cloud-based solutions were considered to offload heavy computations, optimizing clinical deployment despite initial costs. Deploying our lung cancer detection model in clinical settings necessitates seamless technological integration and overcoming various barriers. Ideal cloud-based platforms would manage large datasets and support real-time analysis, integrating smoothly with Electronic Health Records (EHR) for enhanced patient care continuity. Key challenges include compliance with regulations such as HIPAA or GDPR to ensure data privacy and security, gaining clinical acceptance through rigorous training and validation, addressing technological limitations stemming from input data quality, and managing cost implications effectively. By addressing these challenges with robust, scalable solutions, our model can significantly improve diagnostics, reduce healthcare professionals’ workload, and enhance patient outcomes through quicker and more accurate diagnoses.

## Conclusion

This study successfully developed and validated a composite model for lung cancer image classification, leveraging the combined strengths of three preeminent convolutional neural networks—VGG16, ResNet50, and InceptionV3. This integration facilitated a robust and highly accurate model, achieving an overall accuracy of 98.18%. The model demonstrated exceptional precision, recall, and F1-scores across three categories: Benign, Malignant, and Normal cases, underscoring its potential as a reliable diagnostic tool. The use of multiple pre-trained networks allowed the model to extract a diverse set of features from lung images, enhancing its ability to discern subtle patterns indicative of various lung conditions. This approach significantly surpasses the capabilities of traditional single-model methods, providing a more comprehensive analysis with reduced human error and variability. Additionally, the application of advanced metrics like the Matthews Correlation Coefficient and Balanced Accuracy confirmed the model’s efficacy, consistency, and reliability in clinical scenarios. This research contributes to the ongoing evolution of AI in medicine, promising enhancements in the speed, accuracy, and efficiency of disease diagnostics. As AI continues to integrate into clinical workflows, it holds the promise of supporting medical professionals by providing reliable, timely, and accessible diagnostic information, thus improving patient outcomes and the overall efficiency of healthcare services.

Adapting our lung cancer detection model for other cancers involves leveraging pre-trained networks like VGG16, ResNet50, and InceptionV3, which provide robust feature representations learned from diverse datasets. Fine-tuning with specific datasets for new cancers such as breast, skin, or prostate cancer is crucial, necessitating comprehensive datasets that encompass various disease stages, imaging perspectives, and specific condition details. Each cancer type requires tailored adjustments during model training to ensure accurate identification, emphasizing the importance of careful machine learning strategies to distinguish common features from those unique to each condition. Fine-tuning with specific datasets for new cancers such as breast, skin, or prostate cancer is essential, requiring comprehensive datasets that encompass various disease stages, imaging perspectives, and specific condition details. Each cancer type presents unique features that necessitate tailored adjustments during model training to ensure accurate identification. Future research to enhance model generalizability could focus on incorporating diverse datasets covering broader demographics, varied imaging technologies, and different stages of cancer pathology. Integrating multimodal data, including clinical and genetic information alongside imaging data, could enhance diagnostic accuracy. Improving model explainability beyond current tools like Grad-CAM is essential for clinician trust, while optimizing computational efficiency for real-time diagnostics and developing adaptive learning models are critical to maintaining relevance amidst evolving diagnostic technologies.

## Data Availability

The data that support the findings of this study are openly available at https://www.kaggle.com/datasets/adityamahimkar/iqothnccd-lung-cancer-dataset.
