# Correction: Explainable lung cancer classification with ensemble transfer learning of VGG16, Resnet50 and InceptionV3 using grad-cam

**DOI:** 10.1186/s12880-024-01381-7

**Published:** 2024-07-31

**Authors:** Yogesh Kumaran S, J. Jospin Jeya, Mahesh T R, Surbhi Bhatia Khan, Saeed Alzahrani, Mohammed Alojail

**Affiliations:** 1 Department of Computer Science & Engineering, Faculty of Engineering and Technology, JAIN (Deemed-to-be University), Bengaluru, 562112 India; 2 Department of Computer Science and Engineering, SRM Institute of Science and Technology, Ramapuram, Chennai India; 3 School of science, engineering and environment, University of Salford, Manchester, UK; 4 Management Information System Department, College of Business Administration, King Saud University, Riyadh, Saudi Arabia

Correction to: Kumaran S et al. BMC Medical Imaging (2024) 24:176.

10.1186/s12880-024-01345-x

Following the publication of the Original Article, the authors identified an error in the affiliation of one of the authors, Surbhi Bhatia Khan.

**Incorrect**

School of science, engineering and environment, University of Salford, Manchester, UK and Department of Electrical and Computer Engineering, Lebanese American University, Byblos, Lebanon.

**Correct**

School of science, engineering and environment, University of Salford, Manchester, UK.

The affiliations have been updated above and the Original Article has been corrected.

